# Inhibition of choroidal neovascularization by topical application of angiogenesis inhibitor vasostatin

**Published:** 2009-09-18

**Authors:** Shwu-Jiuan Sheu, Youn-Shen Bee, Yi-Ling Ma, Guei-Sheung Liu, Hsiu-Chen Lin, Tse-Liang Yeh, Jau-Chen Liou, Ming-Hong Tai

**Affiliations:** 1 Department of Ophthalmology, Kaohsiung Veterans General Hospital, Kaohsiung, Taiwan; 2 School of Medicine, National Yang-Ming University, Taipei, Taiwan; 3 Department of Biological Sciences, National Sun Yat-Sen University, Kaohsiung, Taiwan; 4 Department of Medical Education and Research, Kaohsiung Veterans General Hospital, Kaohsiung, Taiwan; 5 Department of Mechanical Engineering, National Central University, Chung-Li, Taiwan; 6 Institute of Biomedical Sciences, National Sun Yat-Sen University, Kaohsiung, Taiwan

## Abstract

**Purpose:**

Choroidal neovascularization (CNV) is the leading cause of blindness in patients with age-related macular degeneration (AMD). This study evaluated the inhibitory effect of vasostatin (VS), an endogenous angiogenesis inhibitor, on CNV.

**Methods:**

Anti-angiogenic activity of VS was evaluated in vitro by migration and tube formation assays in human umbilical vein endothelial cells (HUVECs). CNV lesions were induced in Brown Norway rats by fundus argon laser photocoagulation. Beginning one day after CNV induction, rats were treated with eye drops containing 1 μg/ml VS in PBS buffer for three times daily for 20 days. The extent of CNV was examined by flat mount analysis on day 24 or by fundus fluorescein angiography (FAG) on days 21, 28, 35, and 42, respectively. CNV lesions and choroidal vascularity were evaluated by histological analysis. The spatial distribution of topically applied VS in rat eyes was evaluated by immunoblot analysis.

**Results:**

VS inhibited migration and tube formation in HUVECs. Flat mount analysis revealed that, after laser-induced photocoagulation, topical VS application for 20 days significantly reduced CNV lesions. Moreover, serial FAG analysis indicated that a 20 day VS treatment significantly reduced CNV lesions on all subsequent days. Histological analysis revealed attenuated lesions, intact Bruch’s membrane, and reduced choroidal vascularity in VS-treated eyes. Finally immunoblot analysis reveled VS expression in choroids.

**Conclusions:**

Topical VS application suppresses the progression of laser-induced CNV via angiogenesis inhibition and may constitute a therapeutic alternative for excessive neovascularization occurring with ocular diseases.

## Introduction

Choroidal neovascularization (CNV) is the primary cause for vision loss in patients with age-related macular degeneration (AMD) [1]. In patients with the exudative or “wet” form of the disease, choroidal blood vessels grow through Bruch’s membrane into the subretinal space, followed by leakage and accumulation of serum or blood beneath the retinal pigment epithelium (RPE), leading to retinal insult and rapid vision loss [2]. CNV is frequently observed in patients with high myopia, angioid streaks, inflammation, or diabetic retinopathy. Although the molecular basis for CNV is not well understood, an imbalance between pro-angiogenic and anti-angiogenic factors has been proposed in pathologic neovascularization [1,3]. Vascular endothelial growth factor (VEGF) has been identified as a validated target for several retinal vascular diseases, including CNV [4-12]. In turn, this led to the development of VEGF-neutralizing oligonucleotide aptamer (pegaptanib) [4], humanized anti-VEGF monoclonal antibody bevacizumab (Avastin) [5-8], and humanized anti-VEGF monoclonal antibody fragment (ranibizumab) [9-12] for CNV treatment. In particular, ranibizumab and bevacizumab have shown promise in treating CNV and AMD [3-8]. However, these modalities require repeated intravitreal injections, which can result in endophthalmitis or retinal detachment. Thus, effective anti-angiogenesis therapies that could be continuously administrated through a noninvasive route are in demand for long-term control of CNV.

Vasostatin (VS), the N-terminal domain (amino acids 1–180) of calreticulin, is a potent angiogenesis inhibitor isolated from culture supernatants of an Epstein-Barr virus-immortalized cell line [13-15]. Recombinant VS has been shown to inhibit basic fibroblast growth factor (bFGF)-induced or VEGF-induced endothelial cell proliferation in vitro [13,16]. Administration of recombinant VS protein reduced growth of human Burkitt lymphoma and colon carcinoma in murine models [14,15]. In our previous studies, intramuscular VS gene delivery led to elevated VS levels in circulation, profoundly inhibiting tumor growth and CNV in animals [17,18]. Since eye drops are commonly used for long-term treatment of ocular diseases, we evaluated the feasibility of VS for topical application to eyes [16]. VS was found to be stable in an eye drop solution at 4 °C for at least seven days. Moreover, topical VS application eliminated bFGF-induced cornea angiogenesis in rat eyes [16]. Thus, we set out to investigate whether topically applied VS could exert its anti-angiogenic function in internal ocular tissues, such as retina, thereby alleviating CNV induced by laser photocoagulation in rat eyes.

## Methods

### Cell cultures

Human umbilical vein endothelial cells (HUVECs; passage 3 to 6; Sigma Chemical Co; St. Louis, MO) were isolated from umbilical veins as previously described [19] and cultured in Medium 199 (Life Technologies, Gaithersburg, MD) containing 15% fetal calf serum, 20 U/ml porcine heparin (Sigma Chemical Co) and 100 µg/ml endothelial cell growth supplement (Calbiochem-Novabiochem Corp., La Jolla, CA).

### Cell migration assay

Migration assay was performed as previously described [19]. HUVECs were seeded in triplicate in the upper compartment of the chamber (1.2×10^5^ cells in 400 μl) and the lower compartment filled with 200 μl of Dulbecco’s Modified Eagle medium (DMEM; Life Technologies, Gaithersburg, MD) containing 100 ng/ml bFGF (R&D Systems, Inc., Minneapolis, MN) as the chemoattractant. A polycarbonate filter of 8 μm pore size (Nucleopore, Costar, Cambridge, MA), coated with 0.005% gelatin to allow cell adhesion, separated the compartments. After incubation for 2–4 h in a humidified 5% CO_2_ atmosphere at 37 °C, cells on the upper side of the filter were removed. Those that had migrated to the lower side were fixed in absolute methanol, stained with 10% Giemsa solution (Merck KGaA, Darmstadt, Germany), and counted as a mean±standard error of the mean (SEM) per filter under five high power fields from an microscope (Olympus BX40; Olympus Optical Co., Tokyo, Japan; 50×, 100×, 200×, 300×, and 400× magnification).

### Tube formation assay

Tube formation assay was performed as previously described [19]. Briefly, Matrigel (Becton Dickinson, Bedford, MA) was diluted with 4 °C serum-free DMEM to 10 mg/ml. The diluted solution was added to a 24 well plate (200 µl per well) and allowed to form a gel at 37 °C for 30 min. After infection for 24 h, HUVEC (1.5×10^5^ cells per ml) were incubated with Matrigel, then added to each well to incubate for 6–8 h at 37 °C in 5% CO_2_. Under these conditions, endothelial cells form delicate networks of tubes that are detectable within 2–3 h and fully developed after 8–12 h. After incubation, the endothelial tubes were fixed with 3% paraformaldehyde and counted in three to four different lower power fields (25×, 40×, 50×, and 100× magnification). The tube ring was examined daily on an microscope (Olympus CKX41; Olympus Optical Co., Tokyo, Japan; 400× magnification) and recorded.

### Animals

Brown Norway pigmented rats (weight 200–250 g, National Animal Center, Taipei, Taiwan) were used. The animals were handled in accordance with the ARVO Statement for the Use of Animals in Ophthalmic and Vision Research. Rats were anesthetized with an intramuscular injection of 0.15 ml/kg of an equal-volume mixture of 2% lidocaine (Xylocaine; Astra, Astra Södertälje, Sweden) and 50 mg/ml ketamine (Ketalar; Parke-Davis, Morris Plains, NJ).

### Generation of CNV by laser photocoagulation

CNV lesions were induced in rat eyes by laser photocoagulation as previously described [17,20]. Briefly, after the rats were anesthetized, their pupils were dilated with 1% tropicamide (1% Mydriacyl; Alcon Laboratories, Fort Worth, TX). A piece of 18x24 mm standard cover glass (Matsunami Glass, Japan) served as a contact lens. Argon laser (Novus Omni, Coherent, Inc., Palo Alto, CA) irradiation was delivered through a slit lamp (Carl Zeiss, Oberkochen, Germany). Laser parameters were set as follows: spot size of 50 µm, power of 400 mW, and exposure duration of 0.05 s. An attempt was made to break Bruch’s membrane, as clinically evidenced by central bubble formation, with or without intraretinal or choroidal hemorrhage. Eight lesions were created between the major retinal vessels in each fundus.

### Application of VS eye drops

Recombinant VS was purified and applied to eyes as previously described [16]. VS eye drops (1 μg/ml in PBS) were formulated in 1 μg/ml phosphate buffered saline (PBS; Invitrogen, Gland Island, NY; 10 mM phosphate buffer, pH 7.4, 150 mM NaCl) and stored at 4 °C. As with methylcellulose solution, VS is stable in PBS without significant loss of integrity or anti-angiogenesis for at least seven days (data not shown). One day after CNV induction, rats were divided into three groups: untreated (n=8); treated three times daily with 50 μl VS eye drops (1 μg/ml in PBS; n=8); or treated three times a day with 50 μl PBS vehicle (n=8). This was continued for 20 days.

### Quantification of CNV by fluorescein isothiocyanate-dextran labeling

The blood vessels in rat eyes were labeled by perfusion with fluorescein isothiocyanate (FITC)-dextran (2×10^6^ MW; Sigma) as previously described [21,22]. Briefly, after the animal were euthanized by intramuscular injection 0.3 ml/kg of an equal-volume mixture of 2% lidocaine (Xylocaine; Astra) and 50 mg/ml ketamine (Ketalar; Parke- Davis, Morris Plains) on day 24, approximately. The eyes were removed, then fixed for 2 h in 10% phosphate-buffered formalin. After the cornea and lens were removed, RPE-choroid-sclera flat mounts were obtained by hemisecting the eye and peeling the neural retina away from the underlying RPE. The retina was detached and flat mounted onto a slide. The fluorescence in flat mounts was examined by fluorescence microscopy (Olympus BX40), and images digitized using a three-color charge coupled video camera and frame grabber (Olympus DP70). The area of hyperfluorescence associated with each burn – the CNV lesion – was measured by image analysis software (Image J, National Institutes of Health, Bethesda, MD).

### Assessment of choroidal neovascularization by fundus fluorescein angiography

CNV lesions were evaluated by fundus fluorescein angiography (FAG) analysis as previously described [17]. On days 21, 28, 35, and 42 after laser photocoagulation, the CNV lesions were studied by FAG using a digital fundus camera (Visupac 450, Ziess FF450, Carl Zeiss, Oberkochen, Germany). Next, 0.1 ml/kg of 10% fluorescein sodium solution (Fluorescite, Alcon Laboratories, Fort Worth, TX) was injected into the intraperitoneal cavity of anesthetized rats. Late-phase angiograms were obtained 8 min after injection, and digital fundus pictures of bilateral eyes were taken within 1 min. CNV was defined as presence of early hyperfluorescence with late leakage at the site of laser injury. Incidence of CNV was calculated by dividing the number of leaking spots by the number of total laser spots. In addition, the mean area of CNV was measured using image analysis software (Retina Angiography Area Measurement, Heidelberg Engineering, Heidelberg, Germany) by two independent ophthalmologists blinded to the experimental design.

### Histological analysis

After the final round of FAG analysis on day 42, the eyes were enucleated (n=8 rats per group), fixed for 30 min in 4% paraformaldehyde, embedded in OCT (OCT; Sakura, Tokyo, Japan), and sectioned to 10 µm thickness. The frozen sections were stained with hematoxylin and eosin for examination under light microscope (Olympus BX40) or immunofluorescence analysis using 1:50 dilution Von Willebrand factor (vWF) antibodies (anti-vWF, Dako Denmark A/S, Glostrup, Denmark) followed by incubation with FITC-conjugated secondary antibodies. Immunofluorescence analysis was performed under fluorescent microscopy.

### Western blot analysis of VS distribution in ocular tissue

The distribution of topically applied VS in rat eyes was evaluated by western blot analysis. Brown Norway rats (n=4) were treated with VS eye drops (1 μg/ml in PBS; 50 µl; 1 µg/ml) in the right eye and PBS in the left eye three times daily for 14 days. Twelve hours after the final topical application, the animals were euthanized. The eyes were enucleated and dissected to collect the cornea and retinochoroid tissues. Subsequently, the ocular tissues were homogenized with lysis buffer containing 50 mM HEPES, 150 mM NaCl, 1% Triton X-100, 10% glycerol, 1.5 mM MgCl_2_, 1 mM EGTA, 1 mM PMSF, 10 μg/ml aprotinin, 2 μg/ml leupetin, 10 μg/ml pepstatin, 100 mM NaF, 1 mM NaVO_4_, and 10 mM sodium pyrophosphate. This material was then centrifuged at 15,300 xg at 4 °C for 20 min to collect the supernatants. Concentrations of protein supernatants were determined using Coomassie Plus Protein Assay reagent (Pierce Biotechnology, Inc., Rockford, IL). Next, 20 μg protein samples were separated on 10% SDS–PAGE and transferred onto PVDF membranes (Immobilon-P membrane; Millipore Corp., Billerica, MA) for subsequent blotting. The recombinant VS protein was detected by applying 1:1,000 dilution anti-6×His and the samples were allowed to sit overnight at 4 °C. Protein loading was normalized with 1:10,000 dilution anti-β-actin antibody (Sigma-Aldrich Co., St. Louis, MO).

### Statistical analysis

All data are presented as mean±standard deviation of indicated repeats. Differences between groups were analyzed by ANOVA with two-tailed probability. A p value of less than 0.05 was considered significant. Results were representative of at least three independent experiments. All statistical analyses were conducted using SPSS software, version 15.0.1 (SPSS, Inc., Chicago, IL).

## Results

### VS inhibited the migration and tube formation of endothelial cells

**Figure 1:**
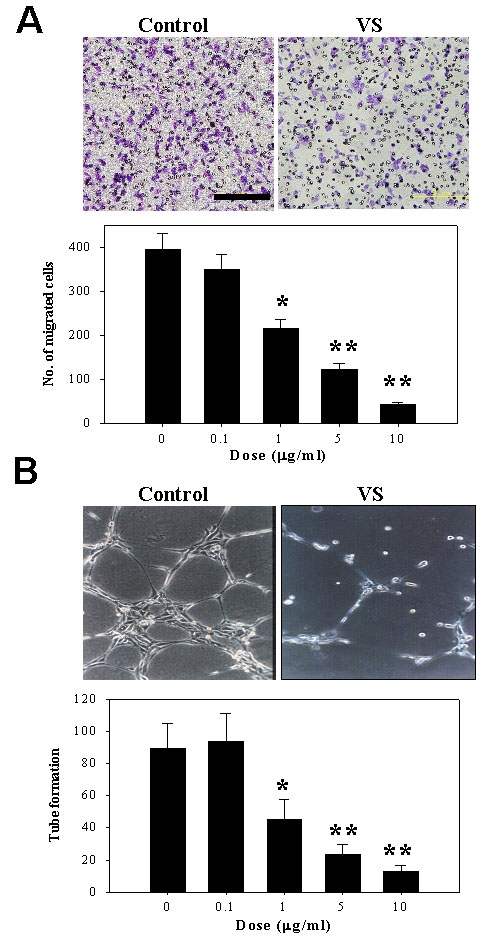
Anti-angiogenic function of VS in endothelial cells. **A**: Effect of VS on migration of HUVECs. VS was added in varying doses, and endothelial cells were placed in a Boyden chamber for 6 h and allowed to migrate toward bFGF. Shown are representative photographs of endothelial migration in control and after treatment with 1 μg/ml VS (left). Cell migration was quantified by counting cells from three high power fields and expressed as mean ± SD of triplicates (right). **B**: Effect of VS on tube formation of HUVECs. Endothelial cells were applied to Matrigel and incubated for 4 h in the presence of VS of varying doses. Representative profiles of the tubular structures in control and 1 μg/ml VS-treated HUVEC are shown (left). Tube formation was quantified by counting the number of rings and expressed as mean±SD from quadruplicates (right). Asterisk (*) represents p<0.05, and double asterisk (**) represents p<0.01.

Application of VS potently inhibited the migration of endothelial cells in a dose-dependent manner (Figure 1A). In addition, application of various doses of VS significantly reduced the capability of endothelial cells to form tube-like structures in Matrigel (Figure 1B). Together, these results indicate that VS perturbed the motility and tube formation of endothelial cells.

### Topical application of VS alleviated the laser-induced CNV lesions

**Figure 2:**
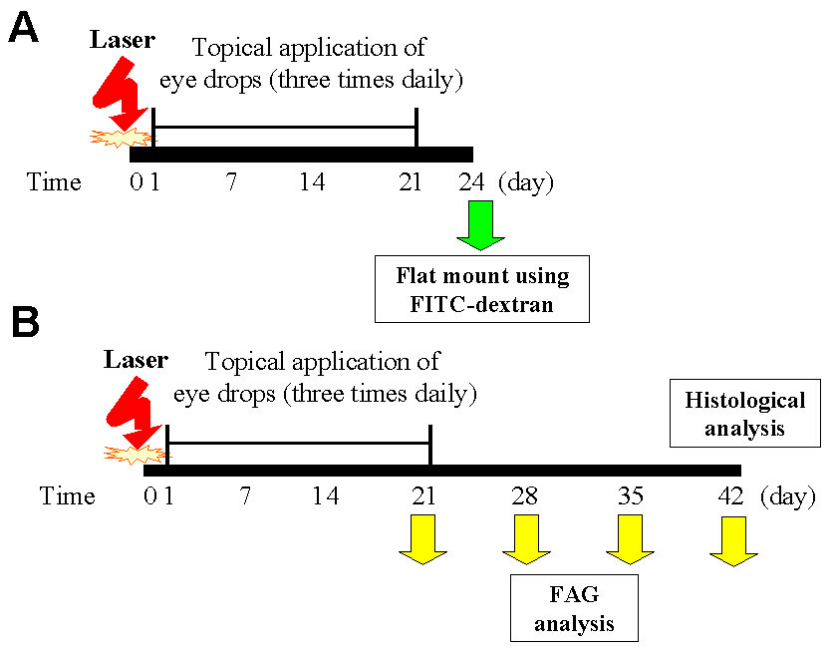
The experimental scheme of topical VS application for laser-induced CNV. **A**: Therapeutic efficacy was assessed by flat mount analysis. CNV was induced by laser photocoagulation on day 0 and validated on day 21 by FAG analysis. The animals (n=8) were euthanized on day 24 to measure CNV extent by flat mount analysis. **B**: In serial FAG analysis, CNV was induced by laser photocoagulation at day 0. From day 1 to 20 after induction, the animals were either left untreated (CNV control group, n=8) or treated with VS eye drops (1 μg/ml in PBS; CNV+VS group, n=8), or PBS vehicle (CNV+PBS group; n=8) three times per day.

**Figure 3:**
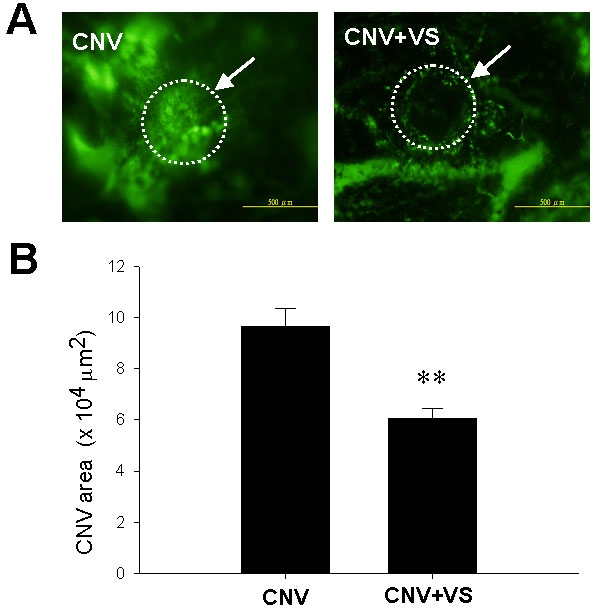
Flat mount analysis of choroidal vascularity in rat eyes after topical VS application as determined by FITC-dextran injection. **A**: FITC-dextran positive blood vessels are presented in choroids of untreated or VS-treated animals. Arrows indicate the laser-induced lesions. **B**: FITC-dextran labeling CNV in untreated or VS-treated eyes are quantified. Data are summarized as mean±SEM (n=8). Double asterisk (**) represents p<0.01. The scale bar equals 200 μm.

The present study employed the model of laser-induced photocoagulation in Brown Norway rats to study the potential of VS eye drops (1 μg/ml in PBS) to treat CNV. The experimental scheme to evaluate the therapeutic efficacy and duration of VS treatment for CNV is depicted in Figure 2. The vascularization in laser-induced CNV lesion in rats after VS treatment was characterized using flat mount analysis after perfusion with FITC-dextran (Figure 3). VS treatment significantly reduced the CNV area (58,964±2,613 μm^2^) compared with untreated CNV (96,764±4,563 μm^2^; p<0.01) and thus was effective in suppressing CNV onset.

### VS treatment diminished the area of CNV lesions by FAG analysis

**Figure 4:**
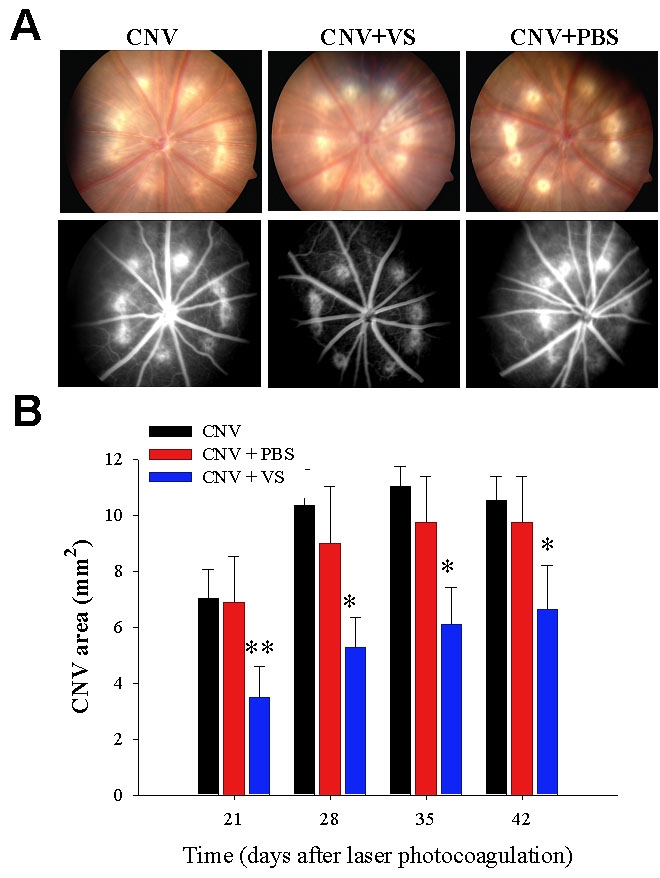
Effect of topical VS application on CNV lesions in rats by FAG. **A**: Representative photographs of fundus microscopy (top panels) and FAG (bottom panels) indicate the anti-angiogenic effect of topical VS application. Top: the laser spots in rat eyes of different group are shown under fundus microscope. Bottom: FAG analysis of CNV lesion in rat eyes by group are shown on day 28. **B**: Effect of topical VS application on the area of CNV lesions in rats are evaluated by FAG. Areas of CNV lesions were determined by FAG examination on days 21, 28, 35, and 42 after laser photocoagulation. Data are summarized as mean±SEM (n=8) and are representative of three experiments. Asterisk (*) represents p<0.05, and double asterisk (**) represents p<0.01.

The therapeutic efficacy of VS eye drops (1 μg/ml in PBS) over time was evaluated by using FAG to analyze laser-induced CNV after VS treatment (Figure 4). VS-treated eyes had less fluorescent dye uptake and extent of CNV (Figure 4A). In addition, CNV lesions areas in VS-treated rats were significantly decreased on day 21 (3.5±1.11 mm^2^ versus 7.01±1.07 mm^2^ and 6.87±2.03 mm^2^ for untreated and PBS-treated eyes, respectively; p<0.01), day 28 (5.27±1.06 mm^2^ versus 10.34±1.3 mm^2^, and 8.99±2.03 mm^2^ for untreated and PBS-treated eyes, respectively; p<0.05), day 35 (6.11±1.33 mm^2^ versus 11.03±0.72 mm^2^ and 9.75±1.62 mm^2^ for untreated and PBS-treated eyes, respectively; p<0.05), and day 42 (3.5±1.11 mm^2^ versus 7.01±1.07 mm^2^ and 6.87±2.03 mm^2^ for untreated and PBS-treated eyes, respectively; p<0.05; Figure 4B). Together, these results indicate that topical VS application attenuated the severity of experimental CNV despite declining efficacy over time.

### Topical VS application alleviated CNV and reduced subretinal neovascularization

**Figure 5:**
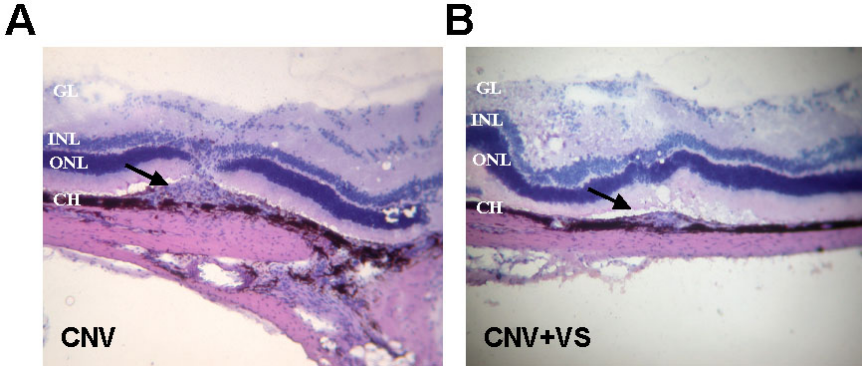
Histological analysis of CNV lesions in rat eyes after topical VS application. After final FAG analysis on day 42, rat eyes were dissected and analyzed by hematoxylin and eosin analysis. VS application resulted in much smaller neovascularization. Arrows indicate the laser-induced CNV lesions. Abbreviations: ganglion cell layer (GL); inner nuclear layer (INL); outer nuclear layer (ONL); choroid (CH). Magnification is 100×.

**Figure 6:**
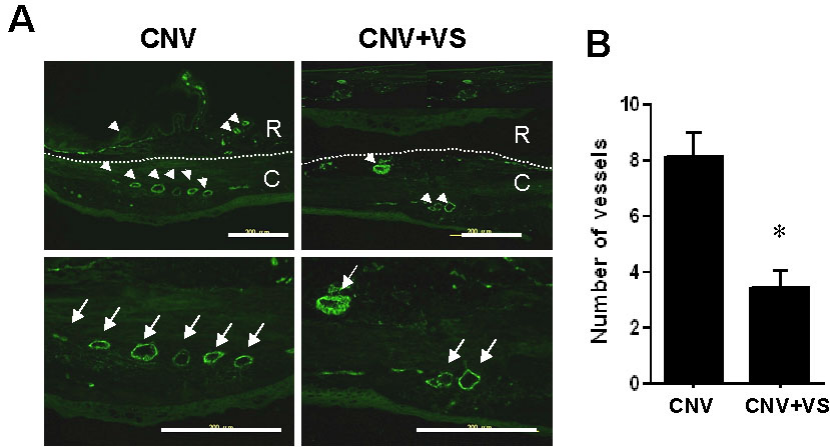
Immunofluorescence analysis of choroidal vascularity in rat eyes after topical VS application. The choroidal vascularity in PBS-treated or VS-treated eyes was examined by immunofluorescence analysis using anti-vWF. **A**: Profile of vWF-positive vessels in choroids (C) and retina (R) of PBS-treated or VS-treated eyes are shown. Arrows indicate CNV by little circles in low power filed (arrowheads) and big circles in high power field (larger arrows). **B**: PBS-treated vWF-positive blood vessels in choroids are nearly twice those of VS-treated eyes. Data are summarized as mean±SEM (n=8). Asterisk (*) indicates p<0.05. The scale bar equals 200 μm.

To elucidate the therapeutic mechanism of VS eye drops (1 μg/ml in PBS), we analyzed rat retinas by histologic and immunofluorescent methods. Hematoxylin and eosin staining showed that laser-induced lesions were definitely smaller and the subretinal tissues relatively intact without neovascularization in VS-treated eyes compared to controls (Figure 5). In addition, examination of blood vessel density revealed that the number of vWF-positive blood vessels was significantly decreased in VS-treated eye drops (1 μg/ml in PBS) compared to controls (8±2.28 versus 3.78±1.16 per high power field; p<0.05; Figure 6). Therefore, topical VS application ameliorated laser-induced lesions and neovascularization, thereby disrupting CNV development.

### Topically applied VS was detected in subretinal tissues

**Figure 7:**
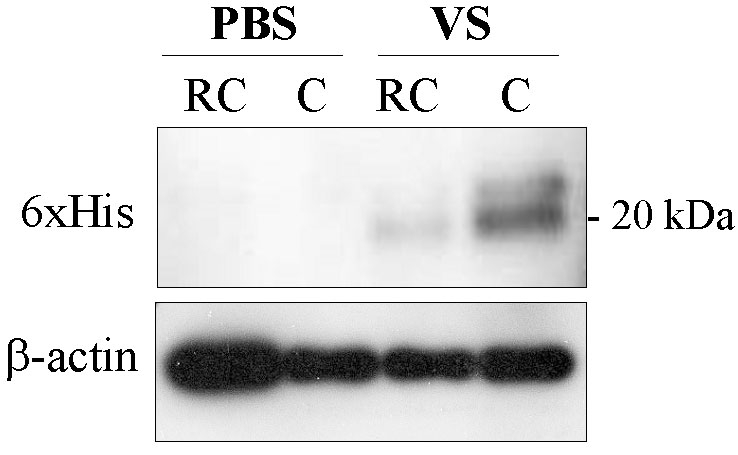
Immunoblot analysis of the distribution of topically applied VS in rat eyes. Western blot analysis reveals that after 14 days of topical VS application, there is greater 6xHis activity and less β-actin in rat cornea (C) and retinochoroid (RC) compared to PBS-treated rat C and RC.

To delineate whether topical VS reached choroids to exert its anti-angiogenic effect, we dissected ocular tissues from VS-treated eyes. Western blot analysis revealed exogenous VS in retinochoroid tissues even though most VS protein accumulated in the cornea (Figure 7). Thus, topically applied VS could reach the choroids.

## Discussion

This study demonstrated that topical VS application suppresses CNV. VS has the following advantages. First, VS protein is highly soluble and stable in solution, making it suitable for eye drop formulation and storage. Second, the potency of VS is greater than that of endostatin or angiostatin [13,14]. Third, the cytotoxicity of VS is highly specific; VS selectively inhibited the proliferation of endothelial cells only, and no other types of cells including retina or iris pigment epithelial cells [16,17]. Finally, topical VS application does not induce inflammation or other adverse effects in animals. Huegel et al. [23] indicated that VS may possess anti-inflammatory capabilities, which may contribute to CNV suppression. Our pilot study using electroretinography also suggest that topical VS application does not alter retinal functions in normal rats (data not shown).

Compared to intraocular injection, topical application is a noninvasive route for drug delivery to the retina with minimal adverse effects. Furthermore, eye drops are used routinely for long-term treatment of glaucoma in clinical practice. Some drugs have been shown to successfully reach the vitreous cavity to treat retinochoroidal diseases [24-26]. Our eye distribution study by western blot analysis suggests that topically applied VS can be transported to the retinochoroid. One probable mechanism is that VS directly penetrates the cornea into the retina. However, the corneal epithelium represents the rate-limiting membrane for water-soluble substances like VS. Alternatively, VS can be transported by a noncorneal route, such as via conjunctiva and sclera. Evidence indicates that the sclera is permeable, even to macromolecules, so that transscleral delivery can enable drug transfer to the posterior segment [27]. Noncorneal routes avoid the lens barrier and the counterflow of aqueous humor [28]. Besides the corneal and noncorneal routes, we could not rule out the possibility that VS is delivered to the posterior ocular segment after systemic reabsorption. In this approach, access to the choroids is feasible owing to the extensive blood flow and highly permeable vessels in the retinochoroid. Future studies are warranted to elucidate the transport mechanism of topical VS therapy.

Because it takes years or even decades for CNV to develop in humans, it is debatable whether a protein or its gene vector should be employed to achieve long-term prevention or suppression of CNV. We have demonstrated the feasibility of intramuscular, polymer-based VS gene therapy for CNV suppression [17]. Though effective and devoid of compliance problems, systemic VS expression may alter physiologic processes, such as wound healing, or by aggravating the cardiovascular diseases. Due to the use of a constitutive promoter, another drawback of gene therapy is the inability to stop the production of gene products when side effects occur. In contrast, despite the short half-life of protein drugs in vivo, topical VS administration has the following advantages: 1) safety via local route; 2) feasibility to extend or terminate the therapy; and 3) flexibility to modify the therapeutic dose. In the present study, the efficacy of VS eye drops (1 μg/ml in PBS) indeed diminished as the experiments went on, probably due to insufficiency of the therapeutic protein. Future experiments should be conducted to investigate whether dose escalation or prolonged treatment could further improve the therapeutic efficacy of VS for CNV.

To our knowledge, this study demonstrates for the first time the potential for topical application of anti-angiogenic protein for CNV. Since none of the experimental animals showed overt adverse effects, VS eye drops (1 μg/ml in PBS) may constitute a novel alternative for CNV. In addition, VS suppresses multiple angiogenic pathways in endothelial cells and may serve as an adjuvant therapy with VEGF-targeting agents such as bevacizumab or ranibizumab for long-term CNV management. Future studies on optimal dosage, therapeutic mechanisms, and pharmacological kinetics of VS are warranted for its ophthalmic application.
